# How Fruitflies Know It's Time for Lunch

**DOI:** 10.1371/journal.pbio.0030332

**Published:** 2005-08-30

To control what you eat and when, your nervous system must coordinate a laundry list of signals: internal signals contain information about energy level, food preferences, and metabolic need, while external signals relay information about the quality of available food, determined by its smell and taste. Scientists studying the fruitfly Drosophila have traced the path of olfactory signals beginning with chemical receptors in the mouth, which set off neurons that signal the antennal lobe of the central nervous system. From here, the electrical stimulation zooms toward the so-called mushroom body, a mushroom-shaped cluster of neurons involved in olfactory processing. Less is known about the gustatory signals, which begin both in the mouth and in the pharynx and aim toward the subesophageal ganglion region of the fly's brain. How olfactory and gustatory signals influence feeding patterns remains murky.

In a new study, Michael Pankratz and Christoph Melcher used genetic analysis to gain insight into the adult and larval neural networks that use taste information to regulate eating. Specifically, they found that several types of neurons responsible for coordinating taste signals express the gene *hugin (hug)*, a gene linked to abnormal eating activity and expressed in only the subesophageal ganglion. By altering *hug* expression, the researchers uncovered the gene's behavioral influence: *hug*-expressing neurons influence a fly's decision to sample new food sources. The researchers also proposed that *hug* proteins play a role in hormone-triggered growth, an important consequence of adequate feeding.

To begin their investigation, Melcher and Pankratz analyzed the DNA from flies with abnormal eating behavior. One group of these flies shared a mutant *klumpfuss (klu)* gene, normally responsible for encoding a protein transcription factor. Because neural transcription factors control production levels of other neural proteins, the researchers used DNA microarrays to compare gene expression in normal flies to that in *klu* mutants. Any *klu*-controlled genes expressed at different levels in *klu* mutants might contain clues about the neural circuitry modulating feeding behavior.

Using microarrays, Melcher and Pankratz discovered that mutant fly larvae overexpress the *hug* gene, which is known to encode at least two neural proteins related to growth signaling. The researchers then investigated which signals influence *hug* expression by exposing larvae to either high or low food levels. Because both starved and sugar-fed flies express little *hug*, the researchers inferred that *hug* levels do not solely signal internal energy requirements but respond to internal and external signals carrying information about the quality of food. The researchers also noted that the finicky *pumpless (ppl)* mutants, which have a feeding defect similar to *klu*, overexpress *hug*.

Behavioral studies confirmed that too much *hug* reduces food intake and leads to stunted growth, while too little stimulates eating. Melcher and Pankratz selected a group of flies and blocked the synapses of their *hug* neurons to inhibit the neurons' activity. In contrast to control flies, which start feeding on a novel food source only after an evaluation phase (they wait a while before initiating feeding), the experimental flies started eating new food right away. These *hug* neurons may help flies decide whether or not to eat a new food source. [Fig pbio-0030332-g001]

**Figure pbio-0030332-g001:**
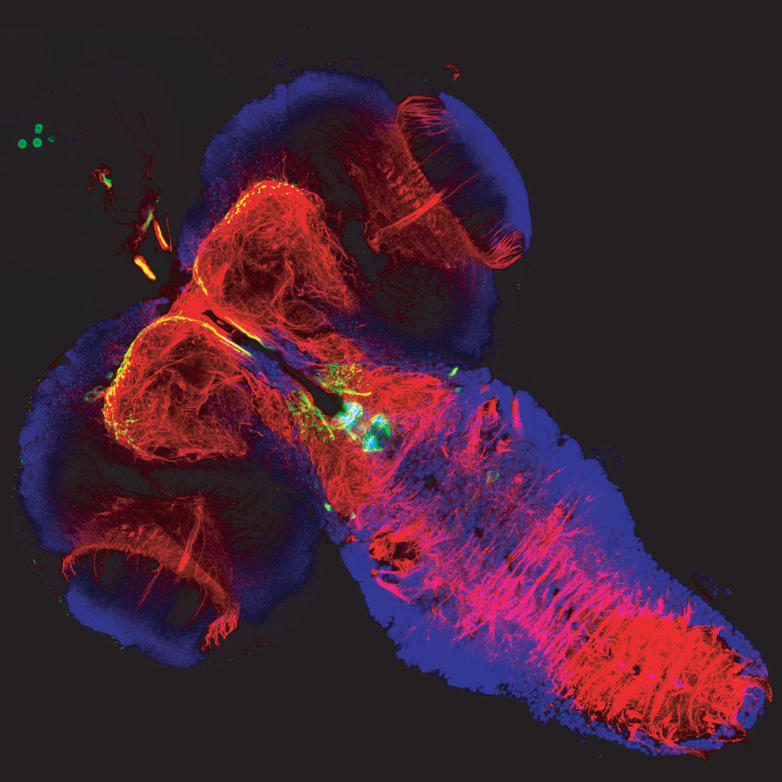
Microarray, neuroanatomical, and biochemical analyses identified taste-sensitive neurons that help regulate feeding behavior in frutifly larvae

Larvae express *hug* in only about 20 neurons, all located in the subesophageal ganglion. The axons of some of these *hug* neurons extend into the ring gland, a crucial metabolism and growth organ in flies. Other axons contact the protocerebrum, a structure close to brain centers for learning and remembering odors. A third set of these axons extend to throat muscles—which is surprising because most subesophageal ganglion neurons have no connection to motor function. All together, these few *hug* neurons can signal structures controlling growth, feeding, and learning and memory.

Besides linking *hug* neurons to brain centers that regulate taste-related feeding behavior, the study also raises questions about how the nervous system prioritizes internal and external signals. How hungry must flies be to overcome taste aversion? How do the competing neural networks of taste and hunger signals decide whether the fly will eat? Future studies pairing behavioral and genetic analysis may begin to reveal answers to these open questions.

